# How Tumor Cells Choose Between Epithelial-Mesenchymal Transition and Autophagy to Resist Stress—Therapeutic Implications

**DOI:** 10.3389/fphar.2018.00714

**Published:** 2018-07-02

**Authors:** Fabrizio Marcucci, Cristiano Rumio

**Affiliations:** Department of Pharmacological and Biomolecular Sciences, University of Milan, Milan, Italy

**Keywords:** EMT, autophagy, mTOR, AMPK, stressor, nutrients, therapy

## Abstract

Tumor cells undergo epithelial-mesenchymal transition (EMT) or macroautophagy (hereafter autophagy) in response to stressors from the microenvironment. EMT ensues when stressors act on tumor cells in the presence of nutrient sufficiency, and mechanistic target of rapamycin (mTOR) appears to be the crucial signaling node for EMT induction. Autophagy, on the other hand, is induced in the presence of nutrient deprivation and/or stressors from the microenvironment with 5′ adenosine monophosphate-activated protein kinase (AMPK) playing an important, but not exclusive role, in autophagy induction. Importantly, mTOR and EMT on one hand, and AMPK and autophagy on the other hand, negatively regulate each other. Such regulation occurs at different levels and suggests that, in many instances, these two stress responses are mutually exclusive. Nevertheless, EMT and autophagy are able to interconvert and we suggest that this may depend on spatiotemporal changes in the tumor microenvironment and/or on duration/intensity of the stressor signal(s). Eventually, we propose a three-pronged therapeutic approach aimed at targeting these three major tumor cell populations. First, cytotoxic drugs that act on differentiated and proliferating tumor cells and which, *per se*, may promote induction of EMT or autophagy in surviving tumor cells. Second, inhibitors of mTOR in order to prevent EMT induction. Third inducers of autophagic cell death (autosis) in order to deplete tumor cells that are constitutively in an autophagic state or are induced to enter an autophagic state in response to antitumor therapy.

## Epithelial-mesenchymal transition (EMT) and autophagy: two different responses to similar stimuli

Tumor cells have evolved two different mechanisms to respond to stress from the tumor microenvironment (TME): epithelial-mesenchymal transition (EMT) (Polyak and Weinberg, 2009; Marcucci et al., 2016) and macroautophagy (hereafter autophagy) (Jiang et al., 2015). EMT endows tumor cells with increased motility, invasiveness, propensity to metastasize, tumor-propagating potential, and resistance to apoptosis and genotoxic stress. Autophagy allows tumor cells to survive under stressful conditions by incorporating cellular material into cytosolic membrane vesicles for catabolic degradation in lysosomes. As such, EMT and autophagy represent two opposite responses to stress from the TME: in one case, escape from an unfriendly environment and dissemination (EMT) and, in the other case, mere survival (autophagy). This might suggest that EMT and autophagy represent tumor cell responses to different forms of stress. However, different stressors such as mechanical stress (Gill et al., 2012; Lien et al., 2013), altered extracellular matrix composition (Neill et al., 2014; Peng et al., 2017), hypoxia (Lu and Kang, 2010; Tan et al., 2016), acidosis (Wojtkowiak et al., 2012; Estrella et al., 2013), genotoxic stress(Wu et al., 2013; Torii et al., 2016), extracellular mediators (Stadler et al., 2013; Jiang et al., 2016; Lou et al., 2016), or endoplasmic reticulum stress (Ma et al., 2014; Shah and Beverly, 2015) can induce proliferating and differentiated tumor cells to undergo either EMT or autophagy. This raises the question as to which mechanism(s) dictate(s) the choice between EMT and autophagy (Gugnoni et al., 2016). In order to address this question, we will first discuss some aspects underlying the induction of EMT and autophagy that are relevant in this setting. For a more detailed discussion on these aspects the reader can refer to several comprehensive reviews (Levine and Kroemer, 2008; Polyak and Weinberg, 2009; Thiery et al., 2009; Jiang et al., 2015).

## Stimuli and pathways that induce EMT in tumor cells

One crucial signaling node involved in the induction of EMT is the mechanistic target of rapamycin (mTOR) (Marcucci et al., 2016). mTOR is part of a signaling pathway that includes, upstream of mTOR, phosphatidylinositol 3-kinase (PI3K) and AKT. mTOR is a Ser/Thr kinase that interacts with several proteins to form two complexes, mTOR complex (mTORC) 1 and 2 (Zoncu et al., 2011). mTOR, as part of mTORC1, is activated by extracellular mediators (Sabatini, 2017). Insulin, for example, induces the sequential activation of PI3K and AKT. AKT then phosphorylates the tuberous sclerosis complex 2, a guanosine triphosphatase (GTPase)-activating protein that leads to the accumulation of the activated, GTP-bound form of Ras homolog enriched in brain (Rheb) which, in turn, activates mTORC1 (Zoncu et al., 2011).

**Figure 1:**
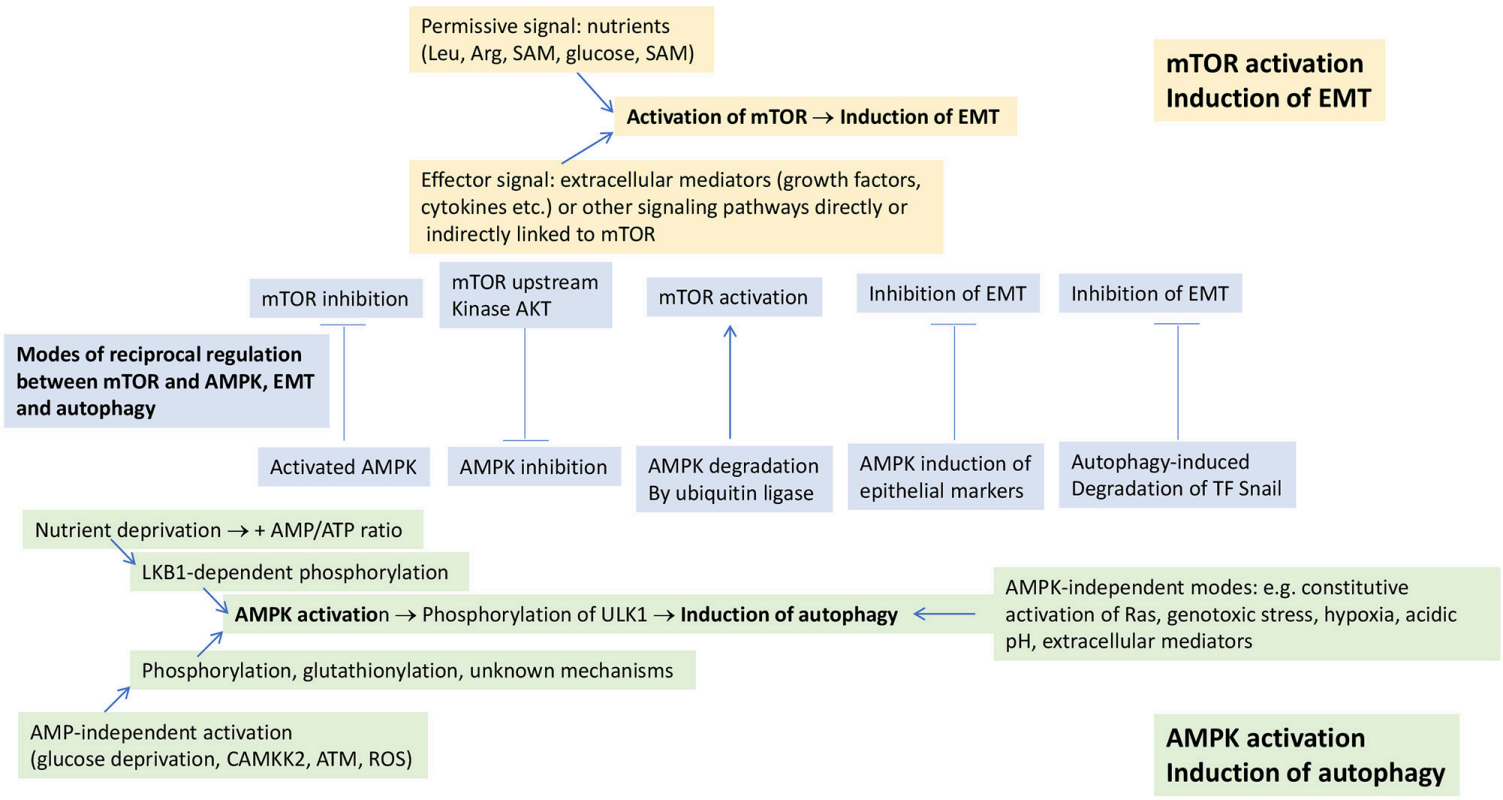
Modes of induction of EMT and autophagy. EMT is induced in response to mTOR activation, which requires two signals for activation: a permissive signal represented by nutrients and an effector signal represented by extracellular mediators and/or other signaling pathways which are directly or indirectly linked to mTOR. Autophagy is induced in response to AMPK activation or to a diverse array of stimuli, independently of AMPK activation. mTOR and AMPK undergo negative, reciprocal regulation (shown in the central part of the figure). Some of the mechanisms underlying the negative, reciprocal regulation between EMT and autophagy that can occur independently of any involvement of mTOR and AMPK are also shown in this part of the figure. See text for further details. ATM, ataxia teleangectasia mutated; CAMKK2, calcium-sensitive kinase calmodulin-dependent protein kinase kinase 2; Rheb, Ras homolog enriched in brain; ROS, reactive oxygen species; SAM, S-adenosylmethionine.

mTOR, however, also responds to amino acids (leucine, arginine, S-adenosylmethionine) and cholesterol (Wang et al., 2015; Saxton et al., 2016; Wolfson et al., 2016; Castellano et al., 2017; Gu et al., 2017), which stimulate the recruitment of mTORC1 to the lysosomal surface, where it interacts with its activator Rheb. mTORC1 is also regulated by cellular glucose levels. The mechanism whereby glucose regulates mTORC1 is distinct from that of amino acids, but the final result is similar, i.e., recruitment of mTORC1 to the lysosomal surface (Efeyan et al., 2015). Thus, a threshold level of nutrients (Saxton and Sabatini, 2017) promote the recruitment of mTORC1 to the lysosomal surface where it interacts with its activator Rheb and other components of a large molecular complex (Dibble and Manning, 2013). As such, mTOR activation is a two-signal system, where nutrients and extracellular mediators exert permissive and effector functions, respectively (Figure 1).

In addition to mTOR activation through PI3K-AKT, other signaling pathways have also been reported to induce EMT in tumor cells (Marcucci et al., 2016). These pathways are, directly, or indirectly, linked to mTOR, suggesting that these signaling pathways induce EMT through their cross-talk with mTOR (Figure 1). Examples of these pathways are RAS-RAF-MEK-ERK (Thorpe et al., 2015), small mother against decapentaplegic (SMAD) (Xue et al., 2012), Wnt (Inoki et al., 2006), Hedgehog (Wang et al., 2012), Notch (Bailis and Pear, 2012), signal transducer and activator of transcription (STAT) 3 (Vogt and Hart, 2011), nuclear factor-κ-light-chain-enhancer of activated B cells (NF-κB) (Lee et al., 2007), focal adhesion kinase (FAK)/SRC (Zhang et al., 2016).

## Stimuli and pathways that induce autophagy in tumor cells

5′ adenosine monophosphate-activated protein kinase (AMPK) is a signaling node that plays for the induction of autophagy a role as important as that of mTOR for EMT. AMPK is a heterotrimer composed of a catalytic (AMPKα) subunit and two regulatory (AMKPβ and AMPKγ) subunits (Herzig and Shaw, 2018). There are two different modes of AMPK activation (Figure 1). First, conditions of nutrient deprivation that elevate the cellular AMP:ATP ratio (Shackelford and Shaw, 2009) and induce binding of AMP to AMPKγ. This promotes the phosphorylation of AMPKα Thr172 by liver kinase B1 (LKB1) and/or inhibits its dephosphorylation by protecting it from phosphatases (Shackelford and Shaw, 2009; Herzig and Shaw, 2018). Second, conditions that are independent of AMP levels. The first of these conditions is glucose deprivation (Zhang et al., 2017; Lin and Hardie, 2018), which leads to the formation, at the lysosomal surface (i.e., the same where mTOR becomes activated), of an AMPK activation complex involving LKB1, AMPK and also components of the mTOR activation complex. Formation of the AMPK activation complex leads to dissociation of mTORC1 from the lysosomal surface. AMPK is then phosphorylated in an LKB1-dependent, but AMP-independent manner (Zhang et al., 2017). AMPK can also be directly phosphorylated on Thr172 in response to calcium flux by the calcium-sensitive calmodulin-dependent protein kinase kinase 2 (CAMKK2) (Hawley et al., 2005; Woods et al., 2005). This kinase activates AMPK in response to stressors like hypoxia (Emerling et al., 2009; Mungai et al., 2011; Lee et al., 2015) or chemotherapeutics through the generation of reactive oxygen species (ROS) (Ji et al., 2010), which may also directly activate AMPK through S-glutathionylation of Cys residues on AMPKα and β (Filomeni et al., 2015). Also ionizing radiation and certain chemotherapeutics like etoposide and cisplatin lead to phosphorylation of AMPKα Thr172 independently of AMP and LKB1, but dependent on signals mediated by ataxia teleangectasia mutated (ATM) (Sanli et al., 2014). AMPK activation under conditions of nutrient sufficiency has also been reported for cytokines and hyperactivated signaling pathways (Herrero-Martín et al., 2009; Kishton et al., 2016).

The existence of AMP-dependent and -independent modes of AMPK activation suggests the possibility that the contemporary presence of elevated AMP levels, low glucose, or other stressors like hypoxia or genotoxic stress (leading to AMP-independent AMPK activation) give rise to additive or synergistic effects. In support of this possibility, it has been shown that, when both are active, the low glucose-sensing pathway and the energy (AMP)-sensing pathway reinforce each other (Lin and Hardie, 2018). In an earlier work, Laderoute et al. (2006) showed that AMPK is activated in response to hypoxia in the presence of glucose sufficiency. The combination of hypoxia and glucose deprivation, however, achieved stronger activation than either stimulus alone. Intuitively, the contemporary presence of stressors and starvation (either glucose and/or other nutrients) appears to be a logical consequence when the stressor(s) lead(s) to a reduction or even zeroing of nutrient supply (e.g., mechanical pressure and hypoxia). This, however, is not necessarily the case. Thus, it has been shown that glucose, a major energetic fuel for tumor cells, diffuses over longer distances than oxygen, thereby allowing to feed, at least transiently, also hypoxic tumor cells (Gatenby and Gillies, 2004). Moreover, tumor cells can also be fed with lactate, the end product of anaerobic glycolysis, the main energetic pathway in hypoxia (Sonveaux et al., 2008). These examples demonstrate that a discrepancy between the presence of stressors and nutrient supply can occur, at least transiently, during tumor growth. Therefore, it appears logical that also AMPK can function as a two-signal system, with AMP-dependent and -independent modes of activation which, whenever acting in concert, lead to additive or synergistic effects in AMPK activation.

Once activated, AMPK phosphorylates a number of downstream substrates that affect energy metabolism and growth, including autophagy induction (Jiang et al., 2015; Mans et al., 2017). Initiation of autophagy requires activation of the Unc-51-like kinase (ULK) complex, which consists of the protein kinase ULK1 and the regulatory proteins Atg13 and FIP200. Activated AMPK phosphorylates ULK1 on at least four residues: Ser467, Ser555, Thr574, and Ser637 (Kim et al., 2011; Herzig and Shaw, 2018). As will be discussed in the following, ULK1 phosphorylation is a point of intersection between opposite effects of mTOR and AMPK on autophagy regulation.

In addition to AMPK-dependent modes, there are also AMPK-independent modes of autophagy induction (Figure 1). Thus, constitutive activation of Ras leading to “autophagy addiction” (Guo et al., 2011), but also stressors like genotoxic stress (Torii et al., 2016), hypoxia (Xue et al., 2016), acidic pH (Wojtkowiak et al., 2012), and extracellular mediators (Jiang et al., 2016) have been reported to induce autophagy in an AMPK-independent manner. It goes beyond the scope of this article to discuss in details the molecular mechanisms underlying AMPK-independent induction of autophagy. Yet, the fact that such pathways exist raises the problem as to which is the relative relevance of AMPK-dependent and -independent mechanisms in the overall induction of autophagy. It has even been argued that the role of activated AMPK would be solely to fine-tune ULK1 activity and subsequent autophagy induction (Jiang et al., 2015). In some instances, however, it has been shown that AMPK activity is a *sine qua non* condition for autophagy induction (Jang et al., 2017). For this reason, we prefer to keep separate AMPK-dependent and -independent modes of autophagy induction.

## The points of discern between induction of EMT and induction of autophagy

While the relevance of AMPK-independent autophagy induction is, at present, difficult to estimate, mTOR and AMPK certainly play pivotal roles in the induction of EMT and autophagy, respectively. On the basis of what has been discussed so far, it appears that EMT is induced by mTOR activation in response to stressors from the TME and in the presence of nutrient sufficiency (Marcucci et al., 2014). On the other hand, autophagy is induced by AMPK activation when one of two situations occur: first, in the presence of nutrient deprivation leading to an elevation of the AMP/ATP ratio; second, in the presence of low glucose and/or stressors that can activate AMPK in an AMP-independent manner. The contemporary presence of starvation and low glucose or other stressors gives rise to additive and/or synergistic effects in AMPK activation.

While nutrient sufficiency is a necessary condition for mTOR activation, nutrient depletion is a sufficient, but not a necessary condition for AMPK activation. The possibility of activating mTOR and AMPK in response to similar stressors and nutrient sufficiency, however, raises the question as to how the choice is made here between mTOR and AMPK activation. We suggest that this may depend on the duration and/or intensity of the stressor signal. Thus, it has been shown that sustained treatment of mammary epithelial cells with transforming growth factor (TGF)-β induces EMT and, subsequently, to loss of the mesenchymal phenotype and induction of autophagy (Jiang et al., 2016). These effects were observed after daily changes of serum-supplemented medium and TGF-β, making other explanations for the induction of autophagy, such as serum consumption and starvation, unlikely. Interestingly, upon further prolongation of treatment, cells underwent apoptosis. This bears strong similarities with an earlier work showing that a growth factor-deprived cell line underwent autophagy allowing it to survive for several weeks but, ultimately, to undergo cell death (Lum et al., 2005). Duration and/or intensity of the stressor signal may also explain some other observations showing that EMT induction in tumor cells can be followed by autophagy or *vice versa*, in response to the same signal (Akalay et al., 2013; Zhu et al., 2014; Whelan et al., 2017).

In addition to duration and intensity of individual stressor signals, it should also be considered that the TME can vary over time and space (Gilkes et al., 2014) and, consequently, also the presence of stressors and nutrient availability. As a consequence of this variability, it appears logical to assume that tumor cells can shift from EMT to autophagy and vice versa. Thus, a shift from EMT to autophagy may occur when nutrient availability becomes inadequate to support EMT; the shift from autophagy to EMT, when nutrient supply resumes after a period of starvation. Antitumor therapy may also impact on this equilibrium given that it represents, *per se*, a stressor (Marcucci et al., 2014), and EMT and autophagy represent two mechanisms of resistance to antitumor therapy (Singh and Settleman, 2010; Shin et al., 2014).

Moreover, it is well known that mTOR and EMT on one hand, and AMPK and autophagy on the other hand, can negatively regulate each other (Dibble and Manning, 2013; Catalano et al., 2015). This occurs at different levels and we will address only some of them here (Figure 1). Thus, activation of mTOR leads to inhibition of autophagy (Fang et al., 2015) through phosphorylation of ULK1 and inhibition of the interaction between ULK1 and AMPK (Kim et al., 2011). Vice versa, activation of AMPK blocks mTOR activation (Inoki et al., 2006; Shi et al., 2012) through phosphorylation of the tuberous sclerosis complex 2 and Rheb inactivation, and through phosphorylation and inhibition of the mTOR binding partner Raptor (Zhao et al., 2017). Inhibition of mTOR has been shown to reverse the mesenchymal phenotype of tumor cells (Chou et al., 2014). Moreover, also AKT, a kinase upstream of mTOR, undergoes a negative, reciprocal regulation with AMPK through AKT-mediated phosphorylation of AMPKα and, vice versa, AMPK-mediated dephosphorylation of AKT (Zhao et al., 2017).

There are also modes of negative, reciprocal regulation of EMT and autophagy that are unrelated to the regulation between AMPK and mTOR (Figure 1). AMPK, for example, was ubiquitinated and degraded by the ubiquitin ligase melanoma-associated antigen (MAGE)-A3/6, and this led to inhibition of autophagy and activation of mTOR (Pineda et al., 2015). Activation of AMPK suppressed EMT by modulating the AKT-mouse double minute 2 homolog (MDM2)-forkhead box O3 (Foxo3) axis, with Foxo3 activation leading to the transactivation of genes encoding epithelial markers and repression of genes encoding EMT-promoting transcriptional regulators (Chou et al., 2014). Autophagy inhibited EMT and promoted mesenchymal-epithelial transition in hepatocytes through degradation of the EMT transcriptional regulator Snail (Grassi et al., 2015). Vice versa, autophagy deficiency stabilized the EMT transcriptional regulator TWIST1 through p62 accumulation (Qiang and He, 2014). EMT induction was also observed upon silencing of the death-effector domain-containing DNA-binding protein (DEDD), and consequent stabilization of Snail and TWIST (Lv et al., 2012). STAT3 inhibited autophagy through inhibition of the expression of the autophagy marker microtubule-associated proteins 1A/1B light chain 3B (LC3) (Gong et al., 2014).

Eventually, autophagy is negatively regulated not only by mTOR, but also by other signaling pathways and nodes that are involved in EMT induction. Inhibition of autophagy by Hedgehog (Jimenez-Sanchez et al., 2012), Wnt (Petherick et al., 2013), and STAT3 (Gong et al., 2014) are examples of pathways negatively regulating autophagy. Whether this occurs as a result of their cross-talk with mTOR is a possibility to be considered in future studies.

## Translational inferences

As already referred to in the beginning, in addition to a mesenchymal-like and autophagic state, tumor cells exist also in a differentiated, proliferating state. We have proposed that mesenchymal-like and autophagic tumor cells can interconvert depending on how conditions in the TME vary over time and space. This implies also the possibility that mesenchymal-like tumor cells or autophagic tumor cells can revert back to a differentiated, proliferating state with predominantly epithelial characteristics. Given that, it appears logical to target, in a therapeutic setting, all of these three populations in order to achieve a tumor cell depletion as complete as possible. In fact, mesenchymal-like tumor cells and autophagic tumor cells, because of their capacity to resist apoptosis and genotoxic stress, are particularly suited to act as reservoirs for the replenishment of proliferating tumor cells once the TME reverts to conditions that are conducive to resume proliferation (Marcucci et al., 2017).

In order to target proliferating tumor cells, the use of one or more cytotoxic drugs that have received regulatory approval over the years seems a logical approach. The choice of the drug(s) will depend on tumor type and therapeutic historical. Drug conjugates for the specific targeting of tumor cells are also becoming available. Cytotoxic drugs, however, may, *per se*, promote resistance in tumor cells and induce them to undergo EMT or autophagy (Marcucci and Corti, 2012).

As regards EMT inhibitors, many compounds with anti-EMT activity have been reported (Marcucci et al., 2016), but in light of the present considerations, the use of mTOR inhibitors appears to be preferred. Some rapalogs have already gained regulatory approval but have shown modest efficacy in tumor therapy (Laplante and Sabatini, 2012). Of greater promise appear catalytic mTOR inhibitors (Fouqué et al., 2015; Rodrik-Outmezguine et al., 2016), which achieve a more complete inhibition of mTOR, but have not yet been approved. In preclinical studies, a catalytic mTOR inhibitor was much more effective than rapamycin in yielding tumor cell apoptosis when combined with an autophagy inhibitor (Fan et al., 2010). mTOR inhibitors, however, can induce autophagy and, consequently, drug resistance (Mitchell et al., 2017). This has led to the synthesis and testing of compounds that act as double mTOR and autophagy inhibitors (Rebecca et al., 2017).

On the autophagy side, the most popular approach has been to use autophagy inhibitors like the lysosomotropic drugs chloroquine or hydroxychloroquine (Pan et al., 2011; Selvakumaran et al., 2013). A considerable number of clinical studies have been started with these compounds (Poklepovic and Gewirtz, 2014). Mixed results have been obtained so far, either as monotherapy, or in combination with other drugs. In some cases negative results have been reported (Rangwala et al., 2014; Wolpin et al., 2014), encouraging results in some other studies (Vogl et al., 2014; Boone et al., 2015; Samaras et al., 2017).

**Figure 2:**
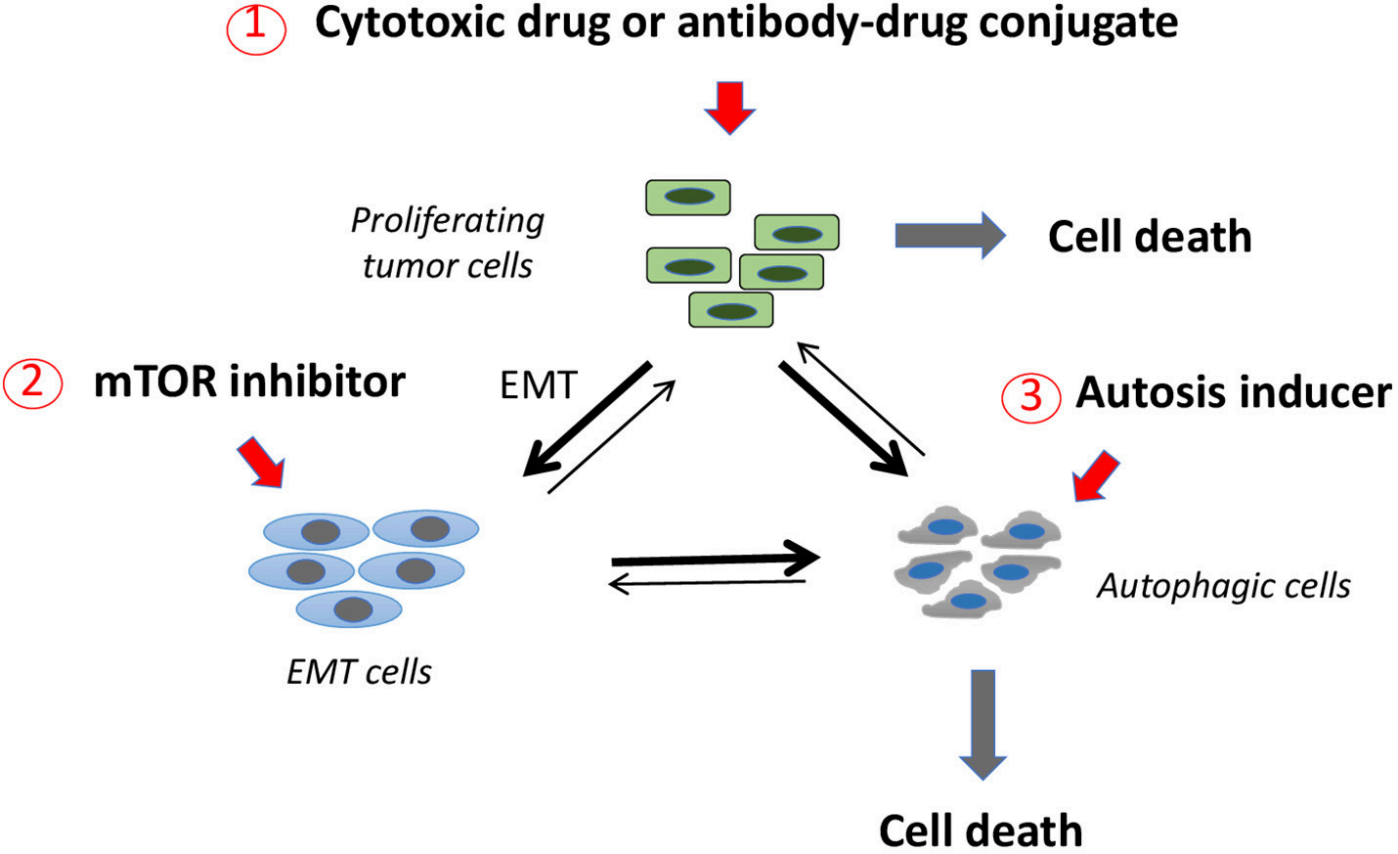
A three-pronged therapeutic approach to target three major tumor cell subpopulations. Proliferating, differentiated tumor cells may be targeted with classical cytotoxic drugs or with antibody-drug conjugates. Tumor cells that come in contact with cytotoxic drug concentrations that are subcytotoxic may respond to this stress by undergoing EMT or autophagy (thick lines). mTOR inhibitors are a second class of drugs that may act preferentially on EMT tumor cells, inducing them to undergo autophagy (thick line). Autophagic cells may be induced toward demise by a third class of drugs, inducers of autophagic cell death (autosis). This therapeutic scheme is expected to act on the three major tumor cell populations leading to their demise and avoiding them to accumulate in one of the two resistant compartments (i.e., EMT or autophagic tumor cells) as a consequence of genotoxic stress.

In several preclinical studies autophagy inhibitors have been shown to promote apoptosis and, when used in combination with other antitumor drugs, improved therapeutic effects were observed (Zeng et al., 2018). In other cases, however, a combination of this kind led to the rescuing of growth-inhibited cancer cells (Oh et al., 2016). Since the effects of autophagy inhibitors still appear unpredictable, we suggest using in combination an mTOR inhibitor as EMT inhibitor and autophagy inducer, and compounds that promote autophagic cell death (autosis) (Liu et al., 2013; Law et al., 2014; Zhai et al., 2014; Shchors et al., 2015; Tomlinson et al., 2015; Yang et al., 2015; Tai et al., 2017). This would allow pushing autophagic tumor cells, whether constitutively present or induced following mTOR inhibition, toward demise. On this basis, we propose to investigate a therapeutic triad in order to target these three major tumor cell populations (Figure 2), i.e., cytotoxic drugs acting on differentiated, proliferating tumor cells; mTOR inhibitors inhibiting EMT induction; autosis inducers promoting death of autophagic tumor cells. Drug combinations of this kind should be tested in appropriate preclinical models for efficacy and unpredictable adverse events and, in case of favorable results, might be considered for testing in the human setting.

## Unresolved questions and concluding remarks

In this article we have addressed the relationship between tumor cell EMT and autophagy. We have proposed a model that allows explaining how tumor cells decide whether to enter one or the other stress response. There are, however, still many open questions that need to be answered. In the following some of those that appear to us most important.

First, we have suggested that signaling pathways or nodes, other than mTOR, that induce EMT in tumor cells do so as a result of their cross-talk with mTOR. While there a number of observations in support of this assumption (Inoki et al., 2006; Lee et al., 2007; Vogt and Hart, 2011; Bailis and Pear, 2012; Wang et al., 2012; Xue et al., 2012; Thorpe et al., 2015; Zhang et al., 2016; Herzig and Shaw, 2018), more conclusive evidence is desirable.

Second, also the role of AMP-dependent and -independent modes of AMPK-dependent autophagy induction and the additive or synergistic effects deriving from the contemporary presence of the two modes requires a more definitive assessment, in spite of indications supporting this possibility.

Third, while mechanisms have been put in place to make EMT and autophagy mutually exclusive events, there are reports showing that phenotypic markers of EMT and autophagy can coexist or that one of the two responses is a necessary condition for the induction of the other(Li et al., 2013; Zhu et al., 2014; Singla and Bhattacharyya, 2017). Singla and Bhattacharyya (2017) have brought evidence active that mTOR signaling is no longer required once tumor cells have undergone EMT and that its inhibition may promote autophagy induction in EMT cells. Clearly, more information is needed to clarify this important point.

In spite of these limitations, our model may represent a useful framework for future work aimed at clarifying the intricate relationship between tumor cell EMT and autophagy (Gugnoni et al., 2016). Eventually, in order to address tumor cell heterogeneity, we have proposed a three-pronged therapeutic approach based on a cytotoxic drug, an mTOR inhibitor and an inducer of autophagic cell death.

## Author contributions

FM and CR contributed to the conception of the work, drafted or revisited it critically, approved the final version, and agreed to be accountable for all aspects of the work.

### Conflict of interest statement

The authors declare that the research was conducted in the absence of any commercial or financial relationships that could be construed as a potential conflict of interest.

## Abbreviations

AMPK: 5′ adenosine monophosphate-activated protein kinase
EMT: epithelial-mesenchymal transition
Foxo3: forkhead box O3
LKB1: kinase liver kinase B1
mTOR: mechanistic target of rapamycin
mTORC: mTOR complex
PI3K: phosphatidylinositol 3-kinase
Rheb: Ras homolog enriched in brain
STAT: signal transducer and activator of transcription
TME: tumor microenvironment
ULK: Unc-51-like kinase.
